# Highlight report: Protection of cholestatic livers by shunting of bile from canaliculi to sinusoids

**DOI:** 10.17179/excli2018-1738

**Published:** 2018-11-19

**Authors:** Tahany Abbass, Walaa Murad, Abdel-latif Seddek

**Affiliations:** 1 Histology Department, Faculty of Medicine, South Valley University, Qena, Egypt; 2 Forensic Medicine and Toxicology Department, Faculty of Veterinary Medicine, South Valley University, Qena, Egypt

## ⁯

Cholestatic liver diseases have been reported to show an anatomically ascending course of disease (Jansen et al., 2017[6]). The first lesions occur in downstream bile ducts followed by damage of the upstream liver parenchyma. In the acute phase after obstruction this may lead to a strong increase of bile acids in bile canaliculi up to concentrations that are toxic to hepatocytes. In the recent issue of Hepatology an interesting mechanism has been reported that prevents deterioration of liver tissue in this acute phase of cholestasis (Ghallab et al., 2018[5]). After bile duct ligation in mice bile acid concentrations increase into cytotoxic range of 0.2-2 mM. Between days 1-3 after bile duct ligation the apical hepatocyte membrane ruptures in individual, dispersed hepatocytes. Within minutes the membrane of the same cell also becomes leaky at the sinusoidal/blood side. This creates a shunt between the bile canaliculus and the sinusoidal blood (Ghallab et al., 2018[5]). As a consequence, bile acids leak from overloaded canaliculi into the blood. Therefore, bile acid concentrations in the canaliculi decrease after shunt formation. In principle, the acute cholestatic liver sacrifices some dispersed individual hepatocytes that later can easily be regenerated in order to save the entire organ.

Analysis of the mechanisms of hepatotoxicity represents an intensively studied field of research (Fickert et al., 2014[2]; Tag et al., 2015[11]; Stöber, 2016[10]; Ghallab, 2017[3]; Ghallab et al., 2016[4]; Vartak et al., 2016[12]). In recent years procedures of mathematical modeling (Schenk et al., 2017[7]; Sezgin et al., 2018[9]; Bartl et al., 2015[1]; Schliess et al., 2014[8]) and advanced imaging techniques such as combined MALDI and two-photon microscopy as applied in the highlighted study (Ghallab et al, 2018[5]) have contributed to a deeper understanding of the pathomechanisms of liver diseases. Although, the here described mechanism of physical shunting of bile salts from high to low concentration areas protects the liver from more severe damage, it should be considered that this leads to higher concentrations in blood with the long term consequence of kidney damage usually in the form of cholemic nephropathy.

## Conflict of interest

There is no conflict of interest.
